# Antimicrobial, Antioxidant and Cytotoxic Activity of Silver Nanoparticles Synthesized by Leaf Extract of *Erythrina suberosa* (Roxb.)

**DOI:** 10.3389/fmolb.2017.00014

**Published:** 2017-03-17

**Authors:** Yugal K. Mohanta, Sujogya K. Panda, Rasu Jayabalan, Nanaocha Sharma, Akshaya K. Bastia, Tapan K. Mohanta

**Affiliations:** ^1^Biochemistry Laboratory, Department of Botany, North Orissa UniversityBaripada, India; ^2^Department of Zoology, North Orissa UniversityBaripada, India; ^3^Department of Life Science, National Institute of TechnologyRourkela, India; ^4^Medicinal Plants and Horticulture Resources, Institute of Bioresources and Sustainable DevelopmentImphal, India; ^5^Free major of Natural Sciences, College of Basic Studies, Yeungnam UniversityGyeongsan, South Korea

**Keywords:** biosynthesis, silver nanoparticle, antimicrobial activity, antioxidant activity, cytotoxic activity

## Abstract

In this experiment, biosynthesized silver nanoparticles (AgNPs) were synthesized using aqueous leaf extract of *Erythrina suberosa* (Roxb.). The biosynthesis of silver nanoparticle was continuously followed by UV-vis spectrophotometric analysis. The response of the phytoconstituents resides in *E. suberusa* during synthesis of stable AgNPs were analyzed by ATR- fourier-transform infrared spectroscopy. Further, the size, charge, and polydispersity nature of AgNPs were studied using dynamic light scattering spectroscopy. The morphology of the nanoparticles was determined by scanning electron microscopy. Current result shows core involvement of plant extracts containing glycosides, flavonoids, and phenolic compounds played a crucial role in the biosynthesis of AgNPs. The antimicrobial activities of silver nanoparticles were evaluated against different pathogenic bacterium and fungi. The antioxidant property was studied by radical scavenging (DPPH) assay and cytotoxic activity was evaluated against A-431 osteosarcoma cell line by MTT assay. The characteristics of the synthesized silver nanoparticles suggest their application as a potential antimicrobial and anticancer agent.

## Introduction

Silver nanoparticles are widely used in pharmaceutical industry in the fabrication of ointments and creams to inhibit burns and wounds related infections (Satyavani et al., 2011). The silver ion has strong inhibitory effect against a number of microorganisms (Mohanta and Behera, 2014). Biological synthesis or green synthesis of nanoparticles is an alternative and eco-friendly method for production of nanoparticles (Firdhouse and Lalitha, 2015; Chung et al., 2016; Nayak et al., 2016). The use of silver nanoparticles both as antimicrobial agent (Majeed et al., 2016) and as a potential drug carrier in treatment of cancer has recently gained considerable attention (Nayak et al., 2016).

Silver nanoparticles can be synthesized using a variety of chemicals and physical methods, involving chemical reduction (Vorobyova et al., 1999; Tan et al., 2002; Yu, 2007), photochemical reduction (Kéki et al., 2000; Pileni, 2000; Sun et al., 2001; Mallick et al., 2005), electrochemical reduction (Sandmann et al., 2000; Liu and Lin, 2004), and heat vaporization (Bae et al., 2002; Smetana et al., 2005). These processes involve several toxic chemicals as reducing agents. Because of using noble metal nanoparticles in areas of human contact (Song and Kim, 2008), there is an emergent need to develop eco-friendly biosynthesis processes that hinders the use of toxic chemicals.

To overcome the complication of toxicity in the synthesis and biological applications, plants or plant extracts have been established to have a leading role in the AgNPs bio synthesis process. Various chemical constituents/phyto-molecules have both protective and reductive activities which are mainly important for the reduction of silver ions adopting natural compounds and reductive enzyme complexes. In recent years, extracellular AgNPs were synthesized using different plant extracts as a potential reducing agent (Ahmed et al., 2016; Mohanta et al., 2016).

*Erythrina suberosa* (Roxb.) is a well-known medicinal plant and used as useful herbal drug in India (Panda, 2014). Its leaves are used for hypotensive, anti-spermatogenic, anti-androgen, anti-gonadotropin, anti-tumor activities, and the bark applied for urinary tract infection (Plants, 1968). *E. suberosa* belongs to the family Fabaceae (sub-family of Leguminasae) and known as the largest flowering plant family of Indian origin.

Till date, no report has present about biosynthesis of AgNPs utilizing an aqueous leaf extract of *E. suberosa*. In the current experiment, extract of *E. suberosa* leaf in a robust aqueous solution of silver nitrate resulted in the reduction of silver ions and the formation of silver nanoparticles. The resulted green-synthesized nanoparticles were examined by ultraviolet-visible spectroscopy (UV-Vis), Transmission Electron microscopy (TEM), Fourier transform infrared (FTIR) spectroscopy, and dynamic light scattering spectroscopy (DLS) to determine their size and charge. The antimicrobial and cytotoxic activities of silver nanoparticles were also evaluated.

## Materials and methods

### Chemicals and reagents

Different chemicals and reagents used during this experiment include; silver nitrate (AgNO_3_), Mueller Hinton agar and Mueller Hinton broth and 3-(4, 5-dimethylthiazol-2-yl)-2, 5-diphenyltetrazolium bromide (MTT), Dulbecco's Modified Eagle's Medium supplemented with 10% fetal bovine serum (FBS), 1% penicillin-streptomycin solution, Bisbenzimide H 33342, and deionized water. All chemicals and reagents are purchased from Sigma Aldrich, India.

### Collection of plants

The plant *E. suberosa* was collected from the Similipal Biosphere Reserve (SBR), India during December, 2014. The SBR is well-known for its natural flora and fauna (Panda et al., 2011). The plant specimen with proper identification was deposited in the post-graduate department of botany, North Orissa University (NOU), Odisha, India. The healthy leaves with clean wash were shade dried and pulverized mechanically followed by percolating to get the homogenous size.

### Preparation of aqueous leaf extract

The shed dried healthy leaves were pulverized separately using mechanical grinder followed by sieving of 40 μm mesh size for further study. Exactly 10 g of leaf powder was added to 100 ml of sterile distilled water and sonicated for 15–20 min. The sonicated extracts were separated by centrifugation (~5,000 rpm) and supernatant was collected for further use. The purified extracts were filtered through Whatman® filter paper and the filtrate was stored at 4°C.

### Qualitative phytochemical analysis

Qualitative phytochemical analysis of the *E. suberosa* extract was performed using the standard experimental procedures to observe the common phyto-constituents. Among the phyto-constituents, alkaloids are analyzed by adopting Mayer's, Wagner's, and Dragendorff's reagents whereas flavonoids by Shinoda alkaline reagent. Similarly, phenolic compounds were tested by lead acetate and alkaline reagent and triterpenes by Liberman-Burchard test. Further, the presence of saponins were determined by foam test and tannins by gelatin test (Parekh and Chanda, 2007; Subashini et al., 2011). The observations of these tests were indicated qualitatively as positive (+) or negative (−).

### Quantitative phytochemical analysis and antioxidant properties

#### TPC determination

Folin-Ciocalteu method was adopted to determine the total amount of phenolics in the leaf extract with little modification (McDonald et al., 2001). All experiments set ups were made in triplicates. The total phenolics content (TPC) was revealed in terms of gallic acid equivalent (GAE) in mg/g sample.

#### TFC determination

The total amounts of flavonoids were determined by modified aluminum chloride method (Chang et al., 2002). All determinations were carried out in triplicates. The total flavonoids content (TFC) was expressed as GAE in mg/g sample.

#### Quantification of radical scavenging activity (DPPH)

To determine the antioxidant activity, 1, 1-diphenyl-2-picryl-hydrazil (DPPH) radical scavenging assay was followed (McDonald et al., 2001). The results were expressed in percentage of radical scavenging activity using butylated hydroxytoluene (BHT) as standard.

### Biosynthesis of silver nanoparticles using leaf extract

The reaction mixture was prepared in a clean glass test tube, by adding 0.5 ml of the aqueous extract and 4.5 ml aqueous solution of 1 mM AgNO_3_. On contrary, 0.5 ml of aqueous leaf extracts with 4.5 ml sterilized deionized water (as control) was kept under dark overnight at room temperature. The color change confirmed the synthesis nanoparticle and solutions with nanoparticle were centrifuged at 10,000 rpm for 45 min (C24-BL centrifuge, REMI, India) with successive washing with deionized water to evacuate any trace of un-utilized phyto-constituents. The remaining pellet was lyophilized and stored for further characterization. The sequence of experimental conditions was revamped for its reproducibility.

### Characterization of silver nanoparticles

#### Ultraviolet-visible absorbance spectroscopy

The bioreduction of silver ions (Ag^+^) into silver nanoparticles (Ag^0^) was monitored in aqueous solution by a UV-Vis spectrophotometer (Lambda 35® PerkinElmer, USA) at regular interval in wavelength ranges between 200 and 1,000 nm.

#### ATR-FTIR spectroscopy

The Attenuated Total Reflection- FTIR spectroscopy analysis of AgNPs was conducted to confirm the promising role of a mixture of phytoconstituents of the plant extracts on the surface alternation and stabilization of biosynthesized AgNPs. The ATR-FTIR was performed using Bruker alpha spectrophotometer (Ettlinger, Germany) with a resolution of 4 cm^−1^. The samples were scanned in the spectral ranges of 4,000–500 cm^−1^ by an average of 25 scans per sample and the result obtained was analyzed through OPUS software.

#### Dynamic light scattering (DLS)

The size and zeta potential (surface charge) of AgNPs were analyzed by Zetasizer (ZS 90, Malvern, UK). The dried samples were adequately diluted with phosphate buffer saline PBS (0.15 M, pH 7.2) prior to investigate in DLS instrument. A scattering angle of 90° was maintained during the assessing of the particle size distribution.

#### Transmission electron microscopy (TEM) study

To further characterize the AgNPs, the Cryo-Transmission Electron microscopy (Cryo-TEM) (Technai™ F30 G2STWIN, FEI, USA) was used to observe the nanodimensional morphology. The synthesized AgNPs were drop coated in a copper grid with mesh size 300 and were observed at 300 kV.

### Antibacterial efficiency of biosynthesized AgNPs

The biosynthesized AgNPs obtained from the leaf extract of *E. suberosa* was tested for its antibacterial potential. The antibacterial activity was determined against Gram-positive bacteria including *Bacillus subtilis* (MTCC 736), *Staphylococcus aureus* (MTCC 737), and Gram-negative bacteria including *Pseudomonas aeruginosa* (MTCC 424), *Escherichia coli* (MTCC 443). The antibacterial activities of the AgNPs were determined by the agar cup and micro broth dilution method using Mueller Hinton medium. Approximate diameter of 6 and 2.5 mm depth wells were made on the exterior of the medium. Exactly 40 μl of synthesized AgNPs were filled in the wells and the same amount of AgNO_3_ solution without plant extracts was served as the control. The antibiotic Gentamycin was used as a positive control for the bacteria. The culture plates were incubated at 37°C for 24 h. After the growth period, the plates were removed and zones of inhibition were measured with Himedia antibiotic scale and the results were tabulated. The AgNPs with zones of inhibition greater or equal to 8 mm diameter were regarded as positive. This study was extended further with broth dilution test. Each experiment was carried out in triplicates. The mean ± *SD* of the zone of inhibitions were taken for calculating the antimicrobial activity of the extracts.

For micro broth dilution method, two-fold serial dilution of the AgNPs was prepared with the MilliQ water 96 well-microtriter plates. Each well of a microtriter plate was inoculated with 190 μL of the test inoculums (0.003 OD) and addition of 10 μL of the AgNPs was made. Only MH broth (190 μL) and AgNPs (10 μL) were taken in a well in order to rectify the absorption variation due to AgNP component present in the reaction mixture. After proper mixing, the plates were sealed with parafilms. The microtriter plates were kept in a shaker-incubator at 37°C for 24 h, and later reading was taken in a iMark™ Microplate Absorbance Reader (Biorad, USA) at 595 nm. The antibacterial activity in micro-broth dilution method was expressed in terms of percent (%) of inhibition.

### Antifungal efficiency of biosynthesized AgNPs

The antifungal activity was tested against *C. albicans* (MTCC 227), *C. kruseii* (MTCC 9215), *T. mentagrophytes* (MTCC 8476), and *C. viswanathii* (MTCC 1929). Potato-dextrose (PD) medium was used to grow the test organism for inoculum. In order to perform the antifungal test, individual well of a microtriter plate was inoculated with the fungal inoculum (190 μL) and AgNPs (10 μL) as test solution. Mixture of 190 μL PD broth and 10 μL AgNPs were kept in a well as control to correct any absorption due to synthesized AgNPs. The standard antifungal drug Clotrimazole was used as a positive control.

### Cytotoxic activity

#### Cell culture

The osteosarcoma cells (A-431, NCCS, Pune, India) were seeded in flask with Dulbecco's Modified Eagle's Medium (DMEM) supplemented with 10% fetal bovine serum (FBS) and incubated at 37°C (5% CO_2_) for 24 h. Post-incubation, the attached cells were trypsinizated for 3–5 min to get the individual cells and centrifuged (800 rpm, 10 min). The cells were counted and distributed in 96-well Enzyme-linked immunosorbent assay (ELISA) plate with 5,000 cells in each well. The plate was incubated for 24 h at 37°C in a 5% CO_2_ atmosphere to allow the cells to form ~70–80% confluence as a monolayer (AshaRani et al., 2009).

#### Cell treatment with AgNPs

Silver nanoparticles strongly reduce the Adenosine Triphosphate (ATP) content of the cell which ultimately cause mitochondrial damage and increases the generation of reactive oxygen species (ROS) in a dose-dependent manner (AshaRani et al., 2009). Hence the toxicity of AgNPs were determined at different concentrations (10, 50, 100, 150, 250 μg/ml) in triplicates and the population of cells were calculated by optical microscopy at 48 h.

#### MTT assay

To detect the cell viability, MTT solution was prepared in growth medium. MTT solution (200 μl) was added to each well of culture and kept for incubation (4–5 h). In the post-incubation period, the MTT solution was removed and DMSO (200 μl) was added to each well under dark condition followed by 15 min incubation. Later the optical density of the formazan product was taken at 595 nm in ELISA reader (Biorad, USA) (Nayak et al., 2016).

### Wound healing assay

The wound healing activity of silver nanoparticles were performed by cell scratch assay (Chang et al., 2002; Yarrow et al., 2004; Chen et al., 2009). Normal fibroblast cell lines (BJ-5Ta) were used to assay the wound healing activity. For this assay, the cells (2 × 10^5^ cells/mL) were seeded in normal cell culture medium DMEM supplemented with 10% phosphate buffer saline (PBS) and M199 medium. After seeding, the cells were incubated for 22–28 h in CO_2_ incubator. With conformation of ~70–80% confluence as a monolayer, the cells were scratched by sharp tips in the mid of the culture well. Thereafter, the ruptured cells were removed by repeated washing with medium. Subsequently the test samples (silver nanoparticles) were added to the scratched wells. During the experiments, Allantoin, a commercial wound healing drug at concentration 50 μg/ml (Sigma Aldrich) was used as +ve control and Hanks' Balanced Salt solution (HBSS) as –ve control. The culture plates were incubated again for 22–24 h until visible of appropriate growth. Later, the cells were fixed and stained to observe the wound healing activity and the photographs were taken in phase contrast microscope.

## Results and discussion

### Phytochemicals analysis and antioxidant activity

#### Phytochemicals analysis of *E. suberosa*

**Figure 1:**
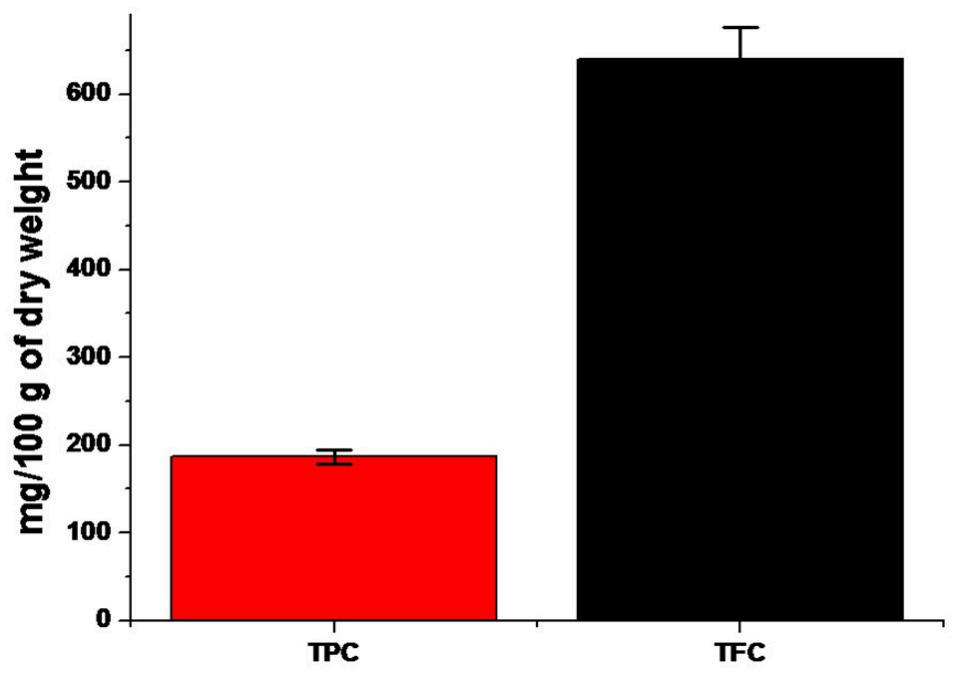
**A comparative bar diagram showing total TPC and TFC contents of ***E. suberosa*****.

The phytochemical screening in both qualitatively and quantitatively of the aqueous extract of *E. suberosa* leaves has been summarized in Figure 1, Tables 1, 2. The investigation revealed the existence different phyto-constituents such as flavonoids, tannins, and phenolic compounds, glycoside and proteins in the leaf extract.

**Table 1:** **Phytochemical screening (qualitative) of ***E. suberosa*** leaf extract**.

**Phytoconstituents**	**Observation results**
Alkaloids	−
Tannins and phenolic compounds	++
Glycoside	+
Flavonoids	+++
Proteins and amino-acids	+
Triterpenoids	−
Steroids and sterols	−

**Table 2:** **Quantitative phyto-chemical constituents of aqueous extract of ***E. suberosa*****.

**Phytochemical constituent**	**mg/100 g dry weight (Mean ± *SD*)**
TPC	186.66 ± 8.32
TFC	640 ± 36.05

Being a valuable medicinal plant, the therapeutic uses and phytochemical investigations of *E. suberosa* are noteworthy. It is used as a potential herbal medicine against different types of body impairments. Our study does not confirm the presence of alkaloids, steroids, and sterols. As per the hypothetical mechanism of biosynthesis of AgNPs, there could be involvement of adequate complex antioxidant enzyme networks (Prasad, 2014). Current investigation result of antioxidant potential of *E. suberosa* offers an affirmative report toward hypothetical mechanism about involvement of antioxidant molecules from the leaf extract in the biogenic synthesis of silver nanoparticles. Previous research study revealed that, plant contains phenolic and flavonoids possess high antioxidant capabilities and hence biosynthesis of nanoparticles (Mohanta et al., 2016).

#### Antioxidant activity of AgNPs

**Figure 2:**
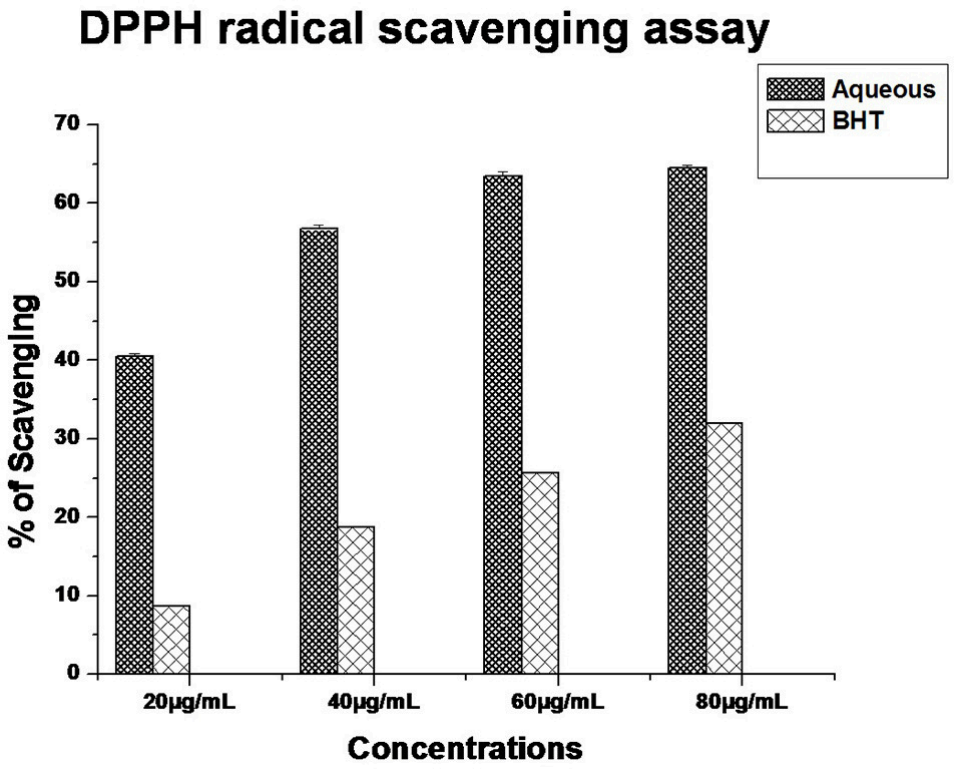
**DPPH radical scavenging capacity of AgNPs**.

The significant antioxidant potential of AgNPs was evaluated by DPPH radical scavenging assay having IC_50_ 30.04 μg/mL (Figure 2). The BHT was used as a standard. Kharat and Mendhulkar (2016) studied the antioxidant activity of synthesized nanoparticles using DPPH assay and observed the antioxidant potentials of photosynthesized nanoparticles (Kharat and Mendhulkar, 2016). They suggested that photosynthesized NPs can be used as a potential free radical scavenger. Priya et al. (2016) studied *in vitro* antioxidant activity of biosynthesized nanoparticles from *P. pinnata* extract and found significant free radical scavenging potential. Patra and Baek (2016) demonstrated presence of strong antioxidant activity in terms of DPPH radical scavenging (IC_50_ 385.87 μg/mL) (Patra and Baek, 2016). The results strongly recommend the application of AgNPs as useful natural antioxidants for health preservation against different oxidative stress associated with degenerative diseases. In fact, antioxidant evaluation is essential for AgNPs before its use *in vivo* models and also human applications.

### Biosynthesis and characterization of silver nanoparticles (AgNPs)

#### Biosynthesis and UV-Vis spectra analysis of AgNPs

**Figure 3:**
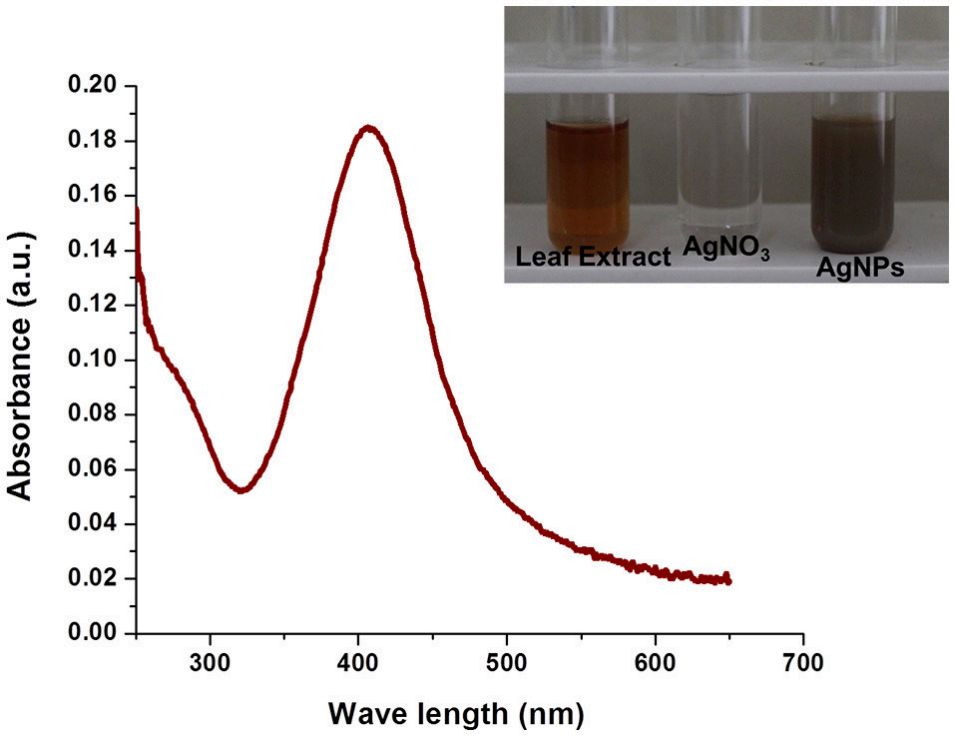
**UV-Vis spectra of AgNPs synthesized by ***E. suberosa*** leaf extracts**. Color change from pale yellow to brown upon synthesis of AgNPs (*In set*).

Bioreduction of silver ions to Ag nanoparticles was evaluated by UV–Vis spectroscopy which is the most simple and indirect method. For this experiment, 9 ml of 1 mM AgNO_3_ solution was taken as the initial amount to which 1 mL of aqueous leaf extract was added and incubated at room temperature (dark condition). After overnight incubation, a visible color change was observed from pale yellow to dark brown. The intensity of the color was increased with increasing in incubation time. In the present study, we observed the appearance of absorption peak at ~428 nm. Previous study reported that AgNPs give absorption peak at 420–450 nm as a result of its surface plasmon resonance (SPR) character (Mohanta et al., 2016; Nayak et al., 2016). In our study, the observed absorption peak at 428 nm further confirms the biosynthesis of Ag nanoparticles (Figure 3).

#### ATR-FTIR spectra analysis of AgNPs

**Figure 4:**
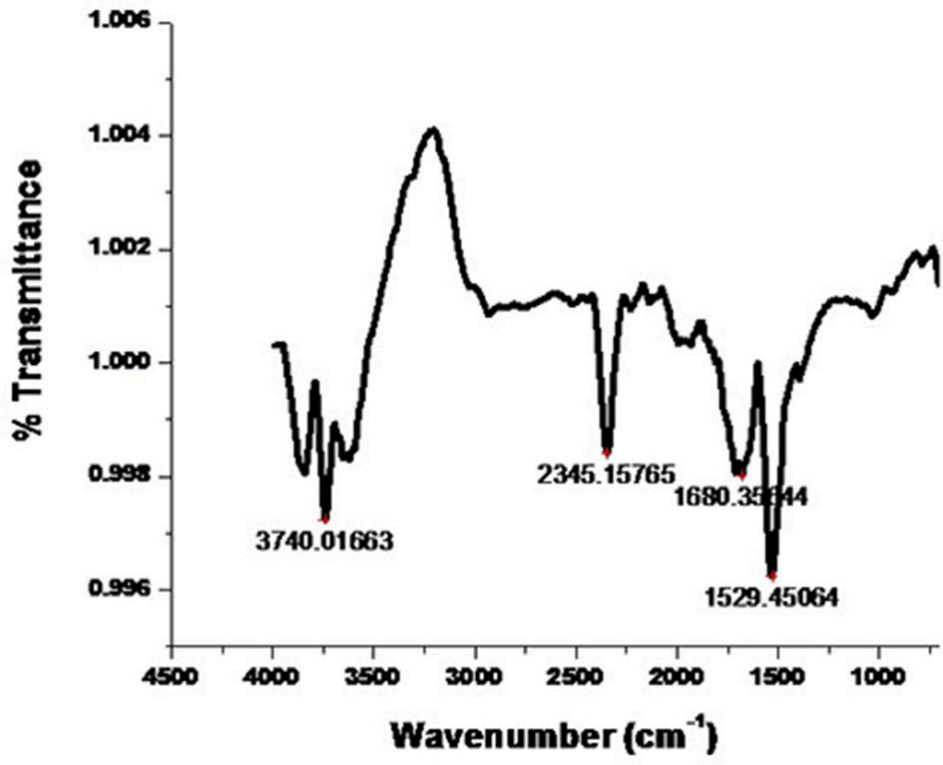
**FTIR spectrum of AgNPs synthesized by ***E. suberosa*** leaf extract**.

The FTIR spectra of the AgNPs confirmed the possible involvement of different functional groups in biosynthesis and stabilization of the nano-particles. It is comprehensible from the spectrum (Figure 4) which indicates the appearance of five prominent absorption peaks at ~3,740, 2,345, 1,680, 1,529, and 1,028 cm^−1^. The strong absorption was found at ~3,740 cm^−1^ indicating the presence of polyphenols due to the binding of silver ion with hydroxyl group which referred to the stretching of OH group or free hydroxyl group. Furthermore, the presence of −C = C− stretch at around 1,680 cm^−1^ confirms the presence of broad range of alkene group in the synthesized nanoparticles. The sharp band at ~1,529 cm^−1^ could possible due to N-O asymmetric stretching indicates the active involvement of nitro compounds. Another medium peak at ~1,028 cm^−1^ indicates the presence of aliphatic amines due to C-N stretching. The presence of characteristic functional groups such as alcohols, aldehydes, flavonoids, phenols, and nitro compounds as phyto-constituents were present in the leaves of *E. suberosa* which participates in the bioreduction process for synthesis of silver nanoparticles.

#### DLS analysis

**Figure 5:**
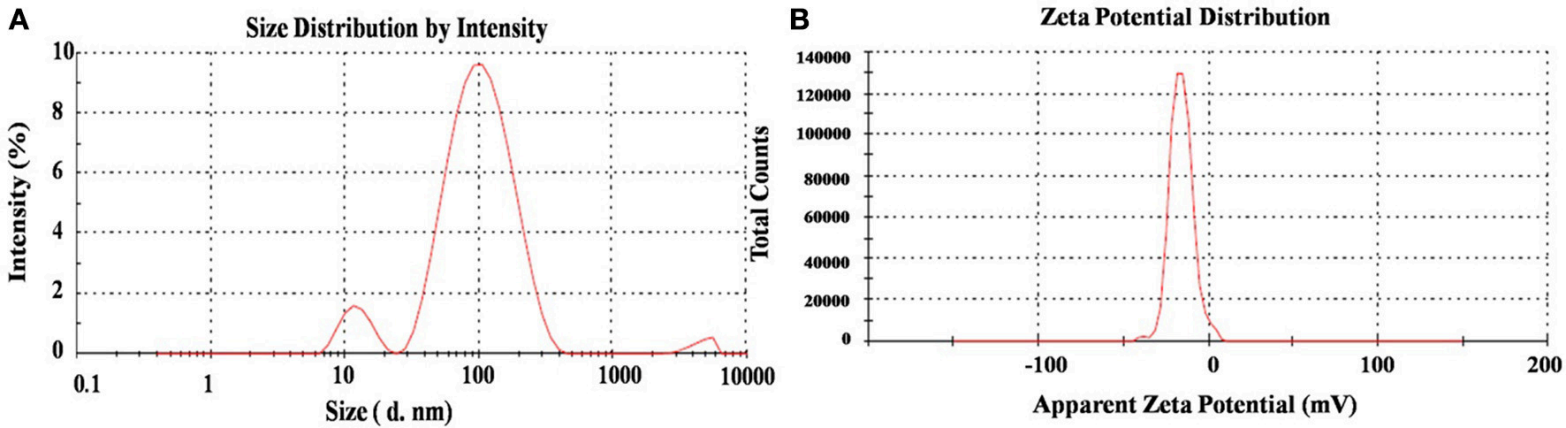
**DLS spectra on (A)** hydrodynamic size distribution and **(B)** Zeta potential (mV) of synthesized AgNPs.

The DLS method revealed the hydrodynamic size and surface charge of AgNPs in aqueous colloidal milieu. With regard to size distributions, it has been found that the AgNPs show an average size of ~12 and 115 nm which confirm its bimodal distribution (Figure 5A). Moreover, the average size and charge of the AgNPs were confirmed to be ~73 nm and −15.8 mV respectively (Figure 5B). The degree of Zeta potential accord initial fluctuation of the particles in the media; but the value is quite adequate to avert further aggregation. Ultimately, the average size and Zeta potential of Ag nanoparticles gives a strong characteristic that can be utilized in biomedical sciences as a biosensor and active drug carrier (Ge et al., 2014).

#### TEM study

**Figure 6:**
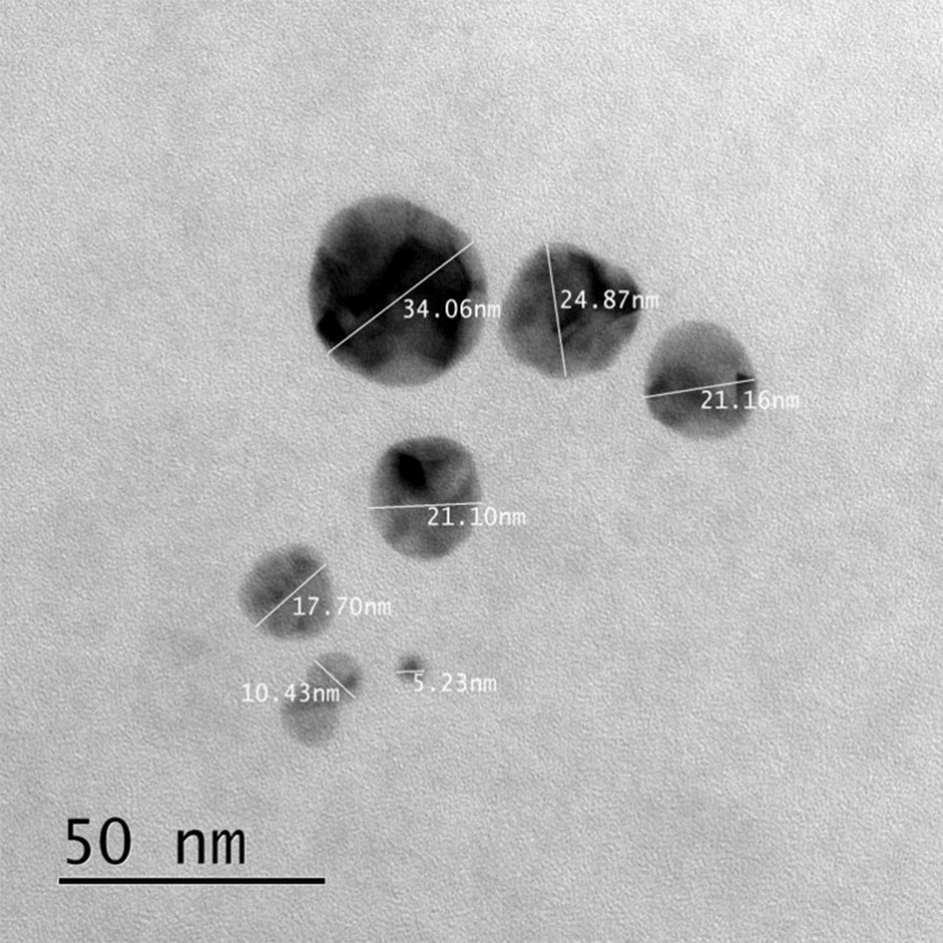
**TEM image of biogenically derived silver nanoparticles**.

Analysis of leaf mediated synthesis of silver nanoparticles by TEM proved the size of AgNPs in the range of nano scale, almost spherically shaped, and has a mean diameter of 15–34 nm (Figure 6). Most of the nanoparticles were approximately circular in shape with smooth edges. In the TEM image, the AgNPs were in physically close contact; but scattered by an adequately uniform distance between particles. Previous study also resulted the biosynthesized silver nanoparticles by *Memecylon edule* leaf extract with circular morphology (Arunachalam et al., 2013).

### Antimicrobial activity of AgNPs

**Figure 7:**
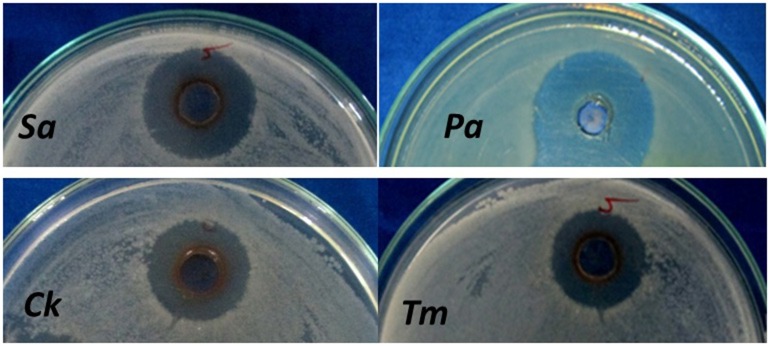
**Antimicrobial activity of AgNPs**. (*Sa, Staphylococcus aureus*; *Pa, Pseudomonas aeruginosa*; *Ck, Candida kruseii*; *Tm, Trichophyton mentagrophytes*).

Pathogenic bacteria and fungi enlisted in Table 3 were taken for preliminary screening of antimicrobial activity using agar cup methods. In agar cup method, zone of inhibitions (ZI) was found maximum against *P. aeruginosa*, and *S. aureus* (Figure 7) whereas no zone of inhibition (ZI) was observed in *E. coli* and *B. subtilis*. Similarly, the inhibition was documented against *C. kruseii* and *T. mentagrophytes* (Figure 7) while no inhibition was observed against *C. albicans* and *C. viswanathii*. For confirmatory antimicrobial potential of AgNPs, the antimicrobial activity against pathogenic bacteria and fungi were carried out through micro broth dilution method and the results in terms of percentage (%) of inhibition were displayed in Table 4.

**Table 3:** **Antimicrobial activity of AgNPs by agar-cup method**.

**Test strains**	**Mean ZI ± *SD* (in mm)**
*Bacillus subtilis*	–
*Staphylococcus aureus*	23 ± 0.8
*Escherichia coli*	–
*Pseudomonas aeruginosa*	24 ± 0.8
*Candida albicans*	–
*C. kruseii*	15 ± 0.8
*C. viswanathii*	–
*Trichophyton mentagrophytes*	16 ± 0.8

**Table 4:** **Antimicrobial activity in broth dilution method**.

**Name of the test strain**	**Growth of inhibition (%) as compared to AgNPs (OD at 595 nm)**
*B. subtilis*	16.27
*S. aureus*	99.26
*E. coli*	28.43
*P. aeruginosa*	95.41
*C. albicans*	36.00
*C. kruseii*	80.27
*C. viswanathii*	74.40
*T. mentagrophytes*	82.27

### Cytotoxic activity against A-431 carcinoma

**Figure 8:**
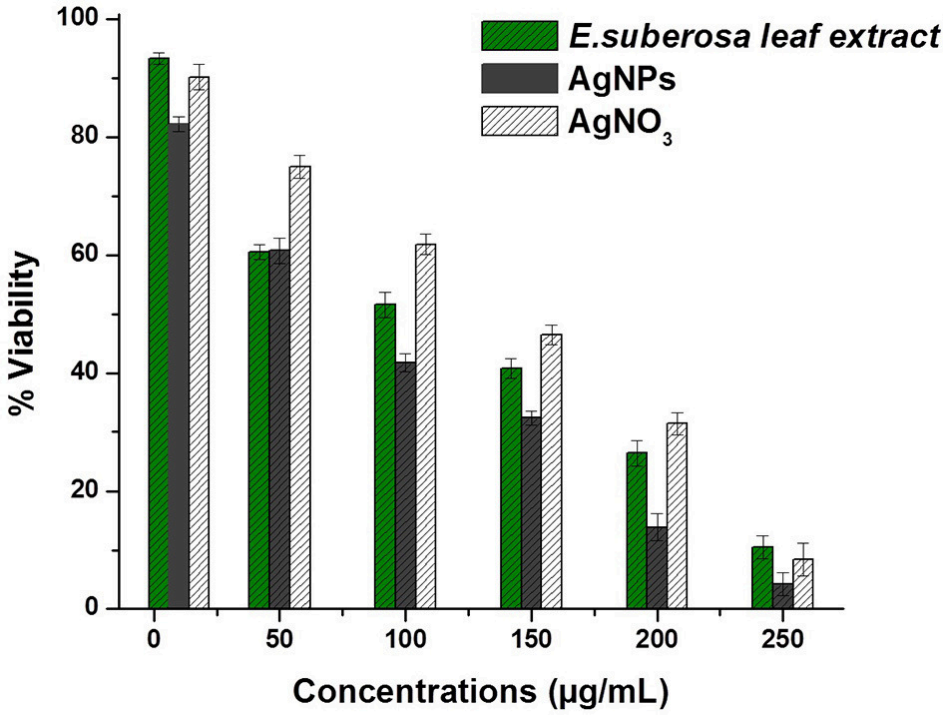
**Cytotoxic activity evaluation against A-431 osteosarcoma cell line**.

Silver nanoparticles (AgNPs) have encouraging application in curative efficacy like wound care, skin cancer and breast cancer (Nayak et al., 2015). Moreover, these are being used as bone cementing and prosthetic materials for fast recovery. In order to support the development of anti-cancer therapy, the biosynthesized silver nanoparticles were taken for cytotoxic study against A-431 osteosarcoma cell line. The cell viability (%) of A-431 cell line post-treatment with biosynthesized silver nanoparticles was determined by studying the MTT assay. The viability of the cells (%) treated with various concentrations of AgNPs has shown in Figure 8. The IC_50_ values were determined to be 106.15 ± 2.6, 74.02 ± 2.4, and 136.73 ± 2.02 μg/mL for leaf extract, AgNPs, and AgNO_3_ respectively. The silver nanoparticles showed excellent anticancer activity against A-431 osteosarcoma cell line with similar IC_50_ values as reported earlier (Park et al., 2011; Gurunathan et al., 2013; Jeyaraj et al., 2013; Krishnaraj et al., 2014; Nayak et al., 2015). This study strongly revealed the significant antiproliferative activity of biosynthesized silver nanoparticles. However, such activity may be due to the synergetic effect of both nano sized silver and the bioactive phytocompounds attached on the surface of the nanoparticles. In depth study will be required to understand the real mechanism behind anticancer activity of the synthesized silver nanoparticles.

### Wound healing assay

**Figure 9:**
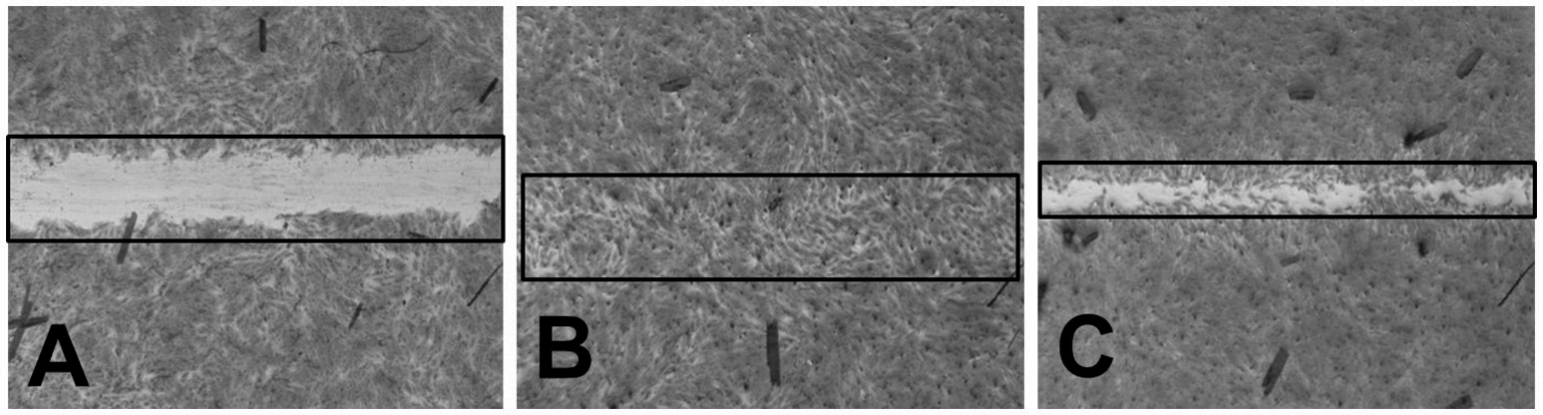
**(A)** BJ-5Ta cells treated with HBSS (−ve control) **(B)** BJ-5Ta cells treated with Allantoin (+ve control) **(C)** BJ-5Ta cells treated with silver nanoparticles.

The wound healing activity of the silver nanoparticles was observed with positive effect (Figures 9A–C). This wound healing potential has strengthened the application of silver nanoparticles as potential medicine. The therapeutic applications of plant-extract-based scaffolds have been earlier reported to heal wounds and reconstitute the skin (Jin et al., 2013). Hence, the current research finding of potential wound healing capacity of plant extract based silver nanoparticles will be a positive additive for the biomedical applications.

## Conclusion

Herein, a sustainable green approach was adopted to synthesize AgNPs using *E. suberosa* leaf extract which produce spherical-shaped AgNPs at ambient conditions without using any harmful reducing or capping agents. The involvement of plant extract in synthesis of AgNPs is confirmed by ATR-FTIR spectroscopy. Moreover, the functional size NPs was synthesized at room temperature. The protocol adopted here for the synthesis of AgNPs can be applied to other metal NPs due to highly oxidized in nature of *E. suberosa* leaf extract and is very likely to be able to reduce metals under different physiological conditions. Moreover, the abundancy and the biomedical applications such as antimicrobial, anti-cancer and wound healing nature of *E. suberosa* leaf extract mediated AgNPs potentially attracts for the up-scaling of metallic nanomaterials to explore various catalytic as well as biomedical applications.

## Author contributions

YKM: conceived the idea and outlined the experimental procedure, performed the major experiments. SKP and RJ: performed the experiments. NS: drafted the manuscript. AB and TM: revised the manuscript.

### Conflict of interest statement

The authors declare that the research was conducted in the absence of any commercial or financial relationships that could be construed as a potential conflict of interest.

## Abbreviations

AgNPs: Silver Nanoparticles
SPR: Surface Plasmon Resonance
AgNO_3_: Silver Nitrate
nm: Nanometer
mm: Millimeter
IC_50_: Inhibitory concentrations
SBR: Similipal Biosphere Reserve
MTT: 3-(4, 5-dimethylthiazol-2-yl)-2,5-diphenyltetrazolium bromide
DPPH: (1, 1-diphenyl-2-picrylhydrazyl)
MTCC: Microbial Type Culture Collection
MH: Mueller-Hinton
PD: Potato Dextrose
DLS: Dynamic light scattering techniques
FE-SEM: Field emission scanning electron microscopy
FT-IR: Fourier transform infrared spectroscopy
UV-Vis: UV Visible spectrophotometer.
